# Mechanism of μ-Conotoxin PIIIA Binding to the Voltage-Gated Na^+^ Channel Na_V_1.4

**DOI:** 10.1371/journal.pone.0093267

**Published:** 2014-03-27

**Authors:** Rong Chen, Anna Robinson, Shin-Ho Chung

**Affiliations:** Research School of Biology, Australian National University, Canberra, ACT, Australia; Xuzhou Medical college, China

## Abstract

Several subtypes of voltage-gated Na^+^ (Na_V_) channels are important targets for pain management. μ-Conotoxins isolated from venoms of cone snails are potent and specific blockers of different Na_V_ channel isoforms. The inhibitory effect of μ-conotoxins on Na_V_ channels has been examined extensively, but the mechanism of toxin specificity has not been understood in detail. Here the known structure of μ-conotoxin PIIIA and a model of the skeletal muscle channel Na_V_1.4 are used to elucidate elements that contribute to the structural basis of μ-conotoxin binding and specificity. The model of Na_V_1.4 is constructed based on the crystal structure of the bacterial Na_V_ channel, Na_V_Ab. Six different binding modes, in which the side chain of each of the basic residues carried by the toxin protrudes into the selectivity filter of Na_V_1.4, are examined in atomic detail using molecular dynamics simulations with explicit solvent. The dissociation constants (*K*_d_) computed for two selected binding modes in which Lys9 or Arg14 from the toxin protrudes into the filter of the channel are within 2 fold; both values in close proximity to those determined from dose response data for the block of Na_V_ currents. To explore the mechanism of PIIIA specificity, a double mutant of Na_V_1.4 mimicking Na_V_ channels resistant to μ-conotoxins and tetrodotoxin is constructed and the binding of PIIIA to this mutant channel examined. The double mutation causes the affinity of PIIIA to reduce by two orders of magnitude.

## Introduction

Voltage-gated sodium (Na_V_) channels play a vital role in cell excitability. Several subtypes of Na_V_ channels such as Na_V_1.3, Na_V_1.7, Na_V_1.8 and Na_V_1.9 are involved in the pain pathway [1], and thus are important targets for pain management. Many polypeptide toxins isolated from venomous animals such as scorpions, cone snails and spiders selectively interfere with the gating mechanisms and ion conduction properties of certain subtypes of Na_V_ channels. These toxins are promising scaffolds for novel pesticides and analgesics [2], [3].

A family of peptide toxins isolated from venoms of cone snails, referred to as μ-conotoxins, are potent and selective blockers of Na_V_ channels. μ-Conotoxins typically consist of 20–25 residues, six of which are cysteines forming three disulfide bridges, known as the inhibitor cystine knot [4], [5]. Some among the most well-characterized μ-conotoxins are GIIIA [6], PIIIA [7], SmIIIA [8] and KIIIA [9]. GIIIA and PIIIA selectively inhibit the skeletal muscle channel Na_V_1.4 with IC_50_ (half-maximal inhibitory concentration) values in the nanomolar range [7], [10], [11], and do not inhibit neuronal channels Na_V_1.7 and Na_V_1.8 at a concentration of 100 μM [12], [13]. On the other hand, SmIIIA and KIIIA inhibit Na_V_1.7 effectively at a concentration of 1 μM [12]. KIIIA was found to ease inflammatory pain in mice [14]. Various derivatives of KIIIA and other toxins have been designed [15]–[20], in an attempt to improve their analgesic effect and pharmaco-chemical properties. In particular, the R14A mutant of KIIIA was found to be 10-fold selective for Na_V_1.7 over Na_V_1.2 and Na_V_1.4 [18], suggesting that in principle selective inhibitors of Na_V_1.7 can be developed from μ-conotoxins.

μ-Conotoxins inhibit Na_V_ channels via a pore-blocking mechanism [21], [22]. On binding to Na_V_ channels, the side chain of a basic residue from a toxin protrudes into the selectivity filter of a channel, thereby inhibiting ion conduction. In the case of GIIIA, an arginine at position 13 has been proposed to be the residue that protrudes into the filter of the receptor channel [6], corresponding to the arginine at position 14 of PIIIA [7], [23]. Extensive mutagenesis experiments performed on PIIIA show that the mutation of each basic residue from the toxin to a neutral amino acid causes the affinity of the toxin for Na_V_1.4 to decrease by 10 to 50 fold, corresponding to a change of +2 to +4 *kT* in the free energy of binding [23], [24]. The change in binding free energy due to any of the charge-changing mutations is lower than the free energy of 6–7 *kT* for a typical salt bridge [25], [26], indicating that none of the mutations completely disrupts the salt bridge between the toxin and the channel pore.

Molecular dynamics (MD) simulations have been fruitfully used to construct model structures of peptide toxins in complex with ion channels such as K^+^ channels and the bacterial Na_V_ channel Na_V_Ab [27]–[29]. Such models, once validated against experimental data, would provide insight into the molecular determinants for the high-affinity and selective binding of a toxin to a channel, which can then be used to guide the design of novel toxins with improved potency and specificity. However, only few computational studies on the binding of μ-conotoxins to Na_V_ channels have been reported [18], [23], [30], [31], largely due to the unavailability of atomic structures for mammalian Na_V_ channels.

Here the binding of μ-conotoxin PIIIA to Na_V_1.4 is examined in atomic detail using MD simulations. Complex structures of all six possible binding modes of PIIIA-Na_V_1.4 are constructed using MD simulations with distance restraints. Similar pattern of interactions is observed in these complexes, suggesting that PIIIA can inhibit Na_V_1.4 with multiple alternative binding modes. The dissociation constant (*K*_d_) of PIIIA-Na_V_1.4 binding measured experimentally is reproduced within 5-fold using potential of mean force (PMF) calculations. A double mutant ([N181R, E172Q]) of Na_V_1.4 mimicking Na_V_ channels resistant to tetrodotoxin (TTX) and μ-conotoxins is constructed. The *K*_d_ value is found to increase by two orders of magnitude after the mutation, suggesting that Na_V_1.4 can be made resistant to PIIIA by this double mutation.

## Methods

### Homology model of Na_V_1.4

A homology model of the human muscle sodium channel Na_V_1.4 is generated using the 1836 amino acid sequence from the protein database of National Center for Biotechnology Information (Reference: NP_000325). Initially, seven partial models are generated from the full sequence on four different structural templates, including PDB IDs 2KBI [32], 2I53 [33], 3RW0 [34], and 3G43 [35], using the homology modeling server SWISS-MODEL [36]. Sequence identify of Na_V_1.4 segments with the different ion channels varies between 15% and 77%. The models are then visualized to determine how they correspond to the transmembrane pore forming domains of the channel. Those parts of Na_V_1.4 external to the pore forming domain are excluded from this study. The crystal structure of the bacterial Na_V_ channel Na_V_Ab [34], the filter of which is in the conductive state suitable for toxins to bind, is used as a template for the final model. Na_V_Ab was used as a template in homology modeling of Na_V_1.4 previously [37]. Since the extracellular loops linking the S5, P1, P2 and S6 helices are significantly longer in Na_V_1.4 than in Na_V_Ab, these loops cannot be modeled reliably on Na_V_Ab. Therefore, the sequence of Na_V_1.4 is then modified to remove these loops (Fig. S1). The modified sequence is then used for homology modeling. As the sequence identity between the modified sequence and Na_V_Ab is low (20–24%), structural similarity between Na_V_1.4 and Na_V_Ab must be assumed. The four homologous domains of Na_V_1.4, referred to as domains I to IV (DI, DII, DIII and DIV), are modeled as discrete subunits. For DII, a second model is constructed on Na_V_Rh, PDB ID 4DXW [38], and the resulting structure is found to be similar to that modeled on Na_V_Ab (root mean square deviation 1.6 Å). This second model appears to align better with the other domains and thus is used in the final model. Each of the four domains generated comprises of the S5, P1, selectivity filter, P2 and S6 structural elements. Ramachandran plots show that more than 97% of the backbone dihedral angles are within favored or allowed regions. To configure the four-fold symmetry of the ion-channel, the four domains of Na_V_1.4 are aligned with Na_V_Ab clockwise [39], [40]. The backbones of these two channels virtually superimpose (Fig. S2). The DEKA ring in the selectivity filter of Na_V_1.4 corresponds to the positions of the equivalent EEEE (Na_V_Ab) and SSSS (Na_V_Rh) rings in bacterial Na_V_ channels. The resulting ion channel has a pore radius of 2.5 Å.

### Molecular dynamics simulations

The Na_V_1.4 channel is embedded in a POPC (2-oleoyl-1-palmitoyl-*sn*-glycero-3-phosphocholine) bilayer built in VMD [41]. The simulation box contains the channel protein, 172 lipids, 68 Na^+^ ions, 59 Cl^−^ ions and 15,743 water molecules. The concentration of NaCl is approximately 0.2 M. The system is equilibrated extensively for 50 ns, and the size of the simulation box evolves to about 85×85×100 Å^3^ at the end of the simulation (Fig. S2). In the first 20 ns of the equilibration during which the inner cavity of the channel becomes fully hydrated, a harmonic restraint is applied to maintain a rigid channel backbone. At 20 ns, a Na^+^ ion in the bulk is manually moved into the filter. Without the presence of this Na^+^ ion the side chain of Lys177 would spontaneously reorient and occlude the ion conduction pathway, consistent with the results of Xia et al. [42]. Subsequently the harmonic restraint is released and the system simulated for a further 30 ns without any restraints. The structure of the channel is stable, as evidenced by the fact that the root mean square deviation of the channel backbone with reference to the initial energy-minimized model fluctuates between 2 and 3 Å over the last 20 ns.

Toxin PIIIA is then added to the system, with its center of mass (COM) 40 Å above the COM of the channel, such that the toxin is not in direct contact with any part of the channel. The solution structure for PIIIA (PDB ID 1R9I) is used [43]. The toxin carries a net charge of +6 *e*, as histidine which adopts predominantly the deprotonated form at the physiological pH of 7.4 is assumed to be neutral. Six Na^+^ ions are removed to maintain charge neutrality. To accelerate the binding of the toxin to the channel, a flat-bottom harmonic distance restraint is applied to the side chain nitrogen atom of a basic residue of the toxin and the center of the carbonyl groups of residues 176 of the channel filter. This method has been successfully applied to several similar systems previously [44]–[46]. The upper boundary of the distance restraint is gradually decreased from 15 to 3 Å over a simulation period of 5 ns, such that the chosen basic residue of the toxin is drawn into the filter. The simulation is continued further for 15 ns with the harmonic restraints removed. To improve sampling each simulation is repeated a second time with a different orientation for the toxin.

MD simulations are performed at 1 atm and 300 K using NAMD 2.9 [47] with periodic boundary conditions applied. The CHARMM36 force field and the TIP3P model for water are used to describe the interatomic interactions [48]–[50]. The switch and cutoff distances for short-range non-bonded interactions are 8.0 Å and 12.0 Å, respectively. The particle mesh Ewald method is used to account for long-range electrostatic interactions, with a maximum grid spacing of 1.0 Å. The SHAKE [51] and SETTLE [52] algorithms are used to keep the bond lengths in the system rigid. A time step of 2 fs is used. Trajectories are saved every 20 ps for analysis. Molecular graphics are generated using VMD [41].

### Umbrella sampling

The umbrella sampling method is used to construct the PMF profile for the unbinding of PIIIA from Na_V_1.4 along the channel axis. Based on the PMF profile the *K*_d_ value for the formation of the toxin-channel complex can be calculated rigorously according to eq. 1 [27]. It is worth noting that *K*_d_ and IC_50_ have been used interchangeably as it may be assumed that the experimentally measured IC_50_ and the true *K*_d_ values are in the same order.

A constant force of 20 kcal mol^−1^ Å^−1^ is applied to pull the toxin out from the binding site, allowing the starting structures of the umbrella windows spaced at 0.5 Å intervals to be generated. The backbones of both the toxin and the channel are maintained rigid during the pulling. The COM of the toxin backbone is restrained to the center of each umbrella window using a harmonic force constant of 30 kcal mol^−1^ Å^−2^. The COM of the channel is at *z* = 0 Å. A flat-bottom harmonic restraint is applied to maintain the COM of the toxin backbone within a cylinder of 8 Å in radius centered on the channel axis. The toxin is allowed to move freely in the plane perpendicular to the channel axis within this cylinder. Each umbrella window is simulated for up to 8 ns until the PMF profile changes by less than 0.5 *kT* in depth over the last 1 ns. The first 1 ns of each window are removed from data analysis. The *z* coordinate of the toxin COM is saved every 1 ps for analysis.

### Data analysis

A salt bridge is assumed to have formed if the distance is less than 4 Å between a side chain oxygen atom from an acidic residue and a side chain nitrogen atom from a basic residue [53]. A hydrogen bond is assumed to be formed if the donor and acceptor atoms (nitrogen or oxygen) are within 3.0 Å of each other and the donor-hydrogen-acceptor angle is ≥150° [54]. The length of a salt bridge is defined as the distance between the COM of the oxygen atoms in the side chain of the acidic residue and COM of the nitrogen atoms in the side chain of the basic residue. The weighted histogram analysis method is used to construct the PMF profile [55]. The *K*_d_ value is derived using the following equation [27]:
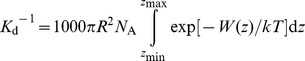
(1)where *R* is the radius of the cylinder (8 Å), *N*_A_ is Avogadro's number, *z*_min_ and *z*_max_ are the boundaries of the binding site along the channel axis (*z*), and *W(z)* is the PMF.

## Results and Discussion

### Outer vestibule of Na_V_1.4

**Figure 1:**
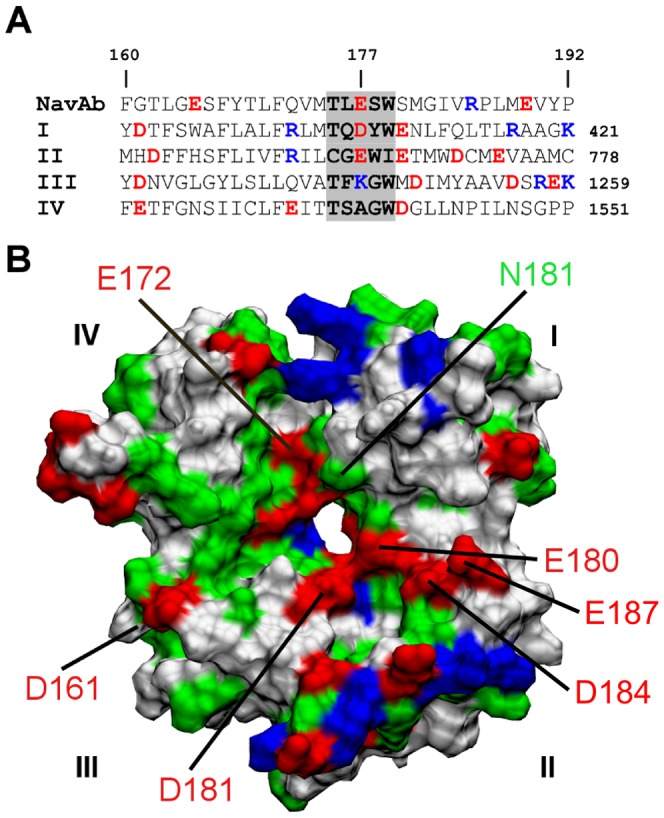
Sequence and structure of Na_V_1.4. (A) Sequence alignment of Na_V_Ab and the four domains (I–IV) of Na_V_1.4 in the pore domain. (B) The outer vestibule of Na_V_1.4 viewed from the extracellular side along the channel axis. For simplicity the numbering of Na_V_Ab is used for Na_V_1.4.

A homology model of the pore domain of Na_V_1.4 is constructed based on the sequence alignment displayed in Fig. 1A (details given in the Methods section). The model of Na_V_1.4 shows that there is an outer ring of acidic residues at positions 180 of DI, DII and DIV and 181 of DIII just outside the filter (Fig. 1B). In addition, two acidic residues at positions 184 and 187 of DII are also in close proximity to the filter. These negatively-charged residues may form strong electrostatic interactions with μ-conotoxins which typically carry several positively-charged basic residues. Thus, our model of Na_V_1.4 is consistent with the high-affinity inhibition of Na_V_1.4 by μ-conotoxins observed experimentally. We demonstrate below that our model of Na_V_1.4 allows both the binding affinity and the selectivity of PIIIA for Na_V_1.4 measured experimentally to be reproduced.

### Selectivity filter of Na_V_1.4

**Figure 2:**
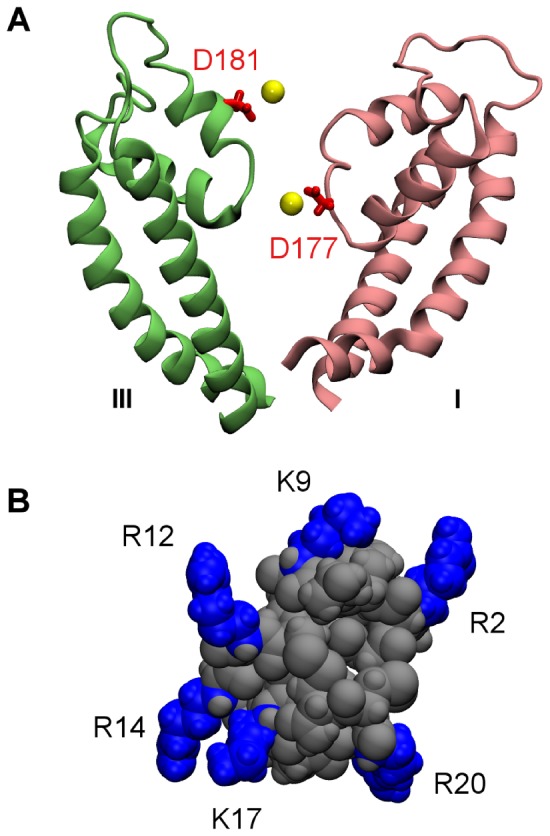
Ion binding site of Na_V_1.4 and structure of PIIIA. (A) The selectivity filter of Na_V_1.4 with two Na^+^ ions (yellow spheres) bound. (B) Molecular structure of PIIIA with the side chains of six basic residues highlighted in blue.

The selectivity filter of Na_V_1.4 carries a DEKA ring (Fig. 1A), as opposed to the EEEE ring found in Na_V_Ab and voltage-gated Ca^2+^ (Ca_V_) channels. This feature of Na_V_1.4 may have important implications for its ion conduction mechanism. After 50 ns of simulation, one Na^+^ ion is present in close proximity to the aspartate residue of the DEKA ring (Fig. 2A). The orientation of Lys177 side chain is stabilized by the two salt bridges it forms with the acidic residues at position 180 of DI and DIV, consistent with a recent study [42]. A second Na^+^ ion is found near the outer ring of acidic residues just outside the filter (Fig. 2A).

### Structure of PIIIA

PIIIA is a small peptide consisting of 22 residues. The primary structure of PIIIA is PcaRLCC-GFOKS-CRSRQ-CKOHR-CC, where Pca and O indicate pyroglutamate and hydroxyproline, respectively. The C-terminus of PIIIA is amidated and the overall charge of PIIIA is +6 *e* at neutral pH resulting from the six basic residues it carries. Fig. 2B shows that these six residues are approximately coplanar and symmetrically distributed around the globular surface of the toxin. This symmetry may be important for the high-affinity binding of PIIIA to Na_V_ channels. For example, we show below that PIIIA can use each of the six basic residues it carries to occlude the selectivity filter of Na_V_1.4, similar to that observed previously for Na_V_Ab [29].

### Binding modes of PIIIA to Na_V_1.4

To predict the most favorable structures of PIIIA in complex with Na_V_1.4, we use MD simulations with distance restraints applied during the first 5 ns, as described in the Methods section. This method allows us to examine all the possible binding modes between the toxin and the channel with affordable computational cost. Two distinct binding modes, in which Lys9 and Arg14 of PIIIA occlude the selectivity filter of Na_V_1.4, respectively, are considered initially. For each binding mode, two simulations started from different initial configuration are performed to improve sampling. Based on the number of salt bridges and hydrogen bonds formed in the toxin-channel complex, the simulation predicting the more favorable structure for each binding mode is considered as representative and discussed in detail below.

**Figure 3:**
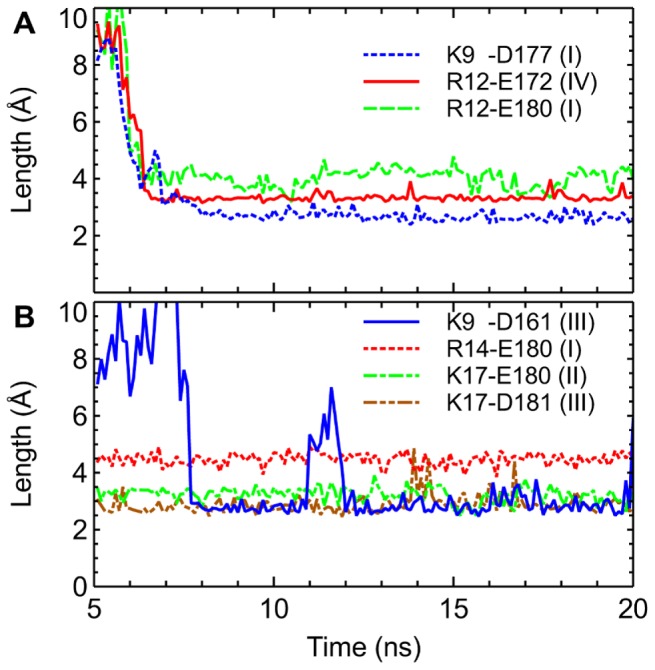
Time evolution of the lengths of the salt bridges in two MD simulations. A distance restraint is applied to Lys9 (A) or Arg14 (B) of PIIIA and the filter of Na_V_1.4 over the first 5 ns of each simulation.

**Figure 4:**
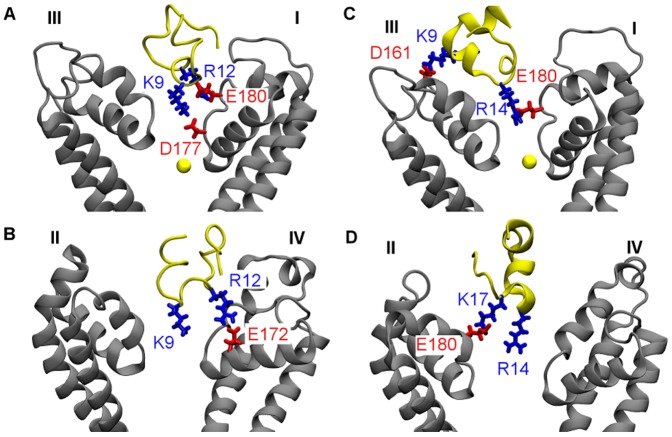
Structures of PIIIA in complex with Na_V_1.4 predicted from MD simulations biased with distance restraints. In two binding modes of PIIIA-Na_V_1.4, Lys9 (A and B) and Arg14 (C and D) of the toxin protrudes into the filter of the channel, respectively. In (A) and (C), the Na^+^ ion inside the filter is shown as a yellow sphere. Toxin backbone is in yellow and channel backbone in grey.

First we look at the simulation in which a distance restraint is applied to Lys9 of PIIIA and the selectivity filter of Na_V_1.4 over the first 5 ns. The distance restraint applied pulls the side chain of Lys9 rapidly into the filter, whereas other residues of the toxin interact favorably with the vestibular wall of the channel. Fig. 3A shows that three salt bridges are formed at 8 ns and remain intact for more than 10 ns during the remaining period of the simulation, suggesting that the complex structure is stable and well equilibrated. One of the three salt bridges (Lys9-Asp177) is located inside the filter, whereas the other two salt bridges are on the vestibular wall (Fig. 4 A and B). The Na^+^ ion inside the filter moves toward the inner cavity of the channel after the binding of PIIIA (Fig. 4A), possibly due to the repelling force from the positive charge carried by Lys9 of PIIIA.

Next we look at the simulation in which Arg14 of PIIIA is drawn into the filter of Na_V_1.4 by using a distance restraint. The position of PIIIA relative to the outer vestibule of Na_V_1.4 is displayed in Fig. 4 C and D. Four salt bridges are formed between the toxin and the channel, of which one is inside the filter and three on the vestibular wall. The salt bridge in the filter, Arg14-Glu180, is relatively weak, with its length longer than 4 Å most of the times (Fig. 3B). The three salt bridges on the vestibular wall, on the other hand, being less than 3.5 Å in length, are relatively strong. Although Arg14 of PIIIA does not form a strong salt bridge with the filter, the position of Arg14 with respect to the filter of the channel appears to be stable.

In the two binding modes discussed above, the toxin-channel complexes are stabilized by three or four salt bridges, whereas the filter residue (Lys9 or Arg14) protrudes deeply into the filter in both modes (Fig. 4). In these two binding modes the toxin interacts intimately with either DI-Glu180 or DII-Glu180, both of which have been shown experimentally to be important for the binding of a closely-related toxin, GIIIA [56]. Similar toxin-channel interactions are observed in all the other four possible binding modes, in which Arg2, Arg12, Lys17 or Arg20 of PIIIA protrudes into the filter of Na_V_1.4, predicted from MD simulations with distance restraints. In all the four modes, the toxin is able to form three or four salt bridges with the channel (Fig. S3), consistent with the Lys9 and Arg14 binding modes we observed (Fig. 4). Thus, the six binding modes of PIIIA-Na_V_1.4 may be of similar energetics. Subsequent PMF calculations are consistent with this proposal.

### PMF profiles

To validate the binding modes of PIIIA-Na_V_1.4 predicted from MD simulations, we construct the PMF profile for the dissociation of the toxin from each complex along the channel axis (*z*). Based on the PMF profile, the corresponding *K*_d_ value is calculated according to eq. 1 and compared to experiment. Due to the limited computational resource available, we derive PMF profiles for the selected two binding modes, in which Lys9 or Arg14 is the filter residue. The Arg14 mode is considered because it has been proposed as the predominant mode in previous experimental studies [23]. The PMF for the Lys9 mode is derived to illustrate that a binding mode equally favorable to the Arg14 mode exists.

**Figure 5:**
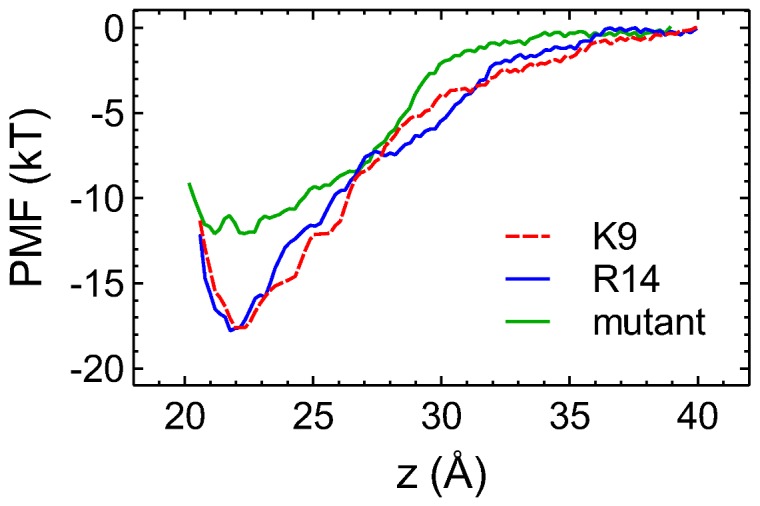
The PMF profiles for the dissociation of PIIIA from Na_V_1.4. The profile for PIIIA binding to a [DI-N181R, DIV-E172Q] double mutant of Na_V_1.4 is also shown. The reaction coordinate *z* is the distance between the centers of mass of toxin and channel backbones along the channel axis. The random errors of the PMF profiles estimated from the bootstrapping method are less than 0.5 *kT* in all cases.

For the Lys9 mode, extra umbrella windows are added at *z* = 23.8 Å and *z* = 25.2 Å to ensure good overlap between umbrella windows. Fig. S4 shows the convergence of the PMF profiles. The converged PMF profiles for the two binding modes in which Lys9 or Arg14 of PIIIA protrudes into the filter of Na_V_1.4 are displayed in Fig. 5. No significant differences in the two profiles are evident, indicating that the two binding modes are of similar energetics.

The depth of the PMF profile for the Lys9 mode is 17.6 *kT*, close to the value of 17.8 *kT* for the Arg14 mode. The *K*_d_ values computed are 300 nM and 150 nM for the Lys9 and Arg14 modes, respectively. Experimentally the *K*_d_ value for the binding of PIIIA to rat Na_V_1.4, which is identical to human Na_V_1.4 in the pore loops, has been estimated to be in the range of 36 nM to 200 nM [12], [23], [30]. Therefore, the *K*_d_ values predicted from PMF calculations do not differ dramatically from those determined experimentally.

We note here that the two PMF profiles we derived are biased. In other words, the umbrella sampling simulations over a limited simulation time only explore a narrow configurational space. In real systems, the toxin would bind with different modes each contributing to an unbiased PMF. The unbiased PMF is reflected in the *K*_d_ value measured experimentally. The fact that the *K*_d_ values derived from the two biased PMF profiles are in good agreement with the experimental *K*_d_ value suggests that the true unbiased PMF profile is similar to the biased PMF profiles we derived (Fig. 5).

Various factors can contribute to the uncertainty in the predicted *K*_d_ values. The accuracy of the force field and molecular models, and the search of configurational space during umbrella sampling largely determine the systematic error, whereas approximating a PMF profile from umbrella sampling simulations can cause random errors. The random errors can be derived, e.g., from bootstrapping methods, while the systematic error can only be estimated by comparing the *K*_d_ values predicted to that determined experimentally. Numerous studies performed on pore blockers of K^+^ channels showed that the systematic error was generally less than 3 *kT* [27], [28]. However, the systematic error for the PMF profiles in Fig. 5 could be higher, due to the uncertainty in the model of the channel. Also, the hetero-tetrameric nature of Na_V_1.4 poses difficulties in the sampling of the configurational space. Nevertheless, the overall error for the PMF profiles of PIIIA-Na_V_1.4 is found to be low, possibly because errors from different sources cancel out.

### A Na_V_1.4 double mutant

**Figure 6:**
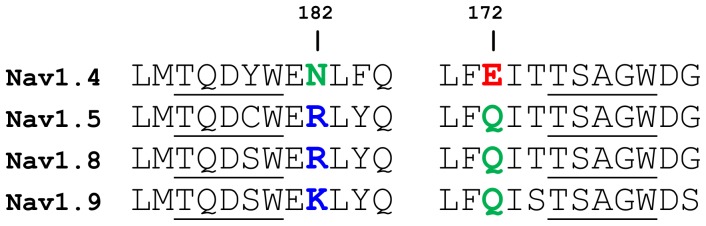
Sequence alignment of Na_V_1.4 and TTX-resistant channels. The selectivity filter region of DI (left) and DIV (right) is shown.

Several isoforms of Na_V_ channels (Na_V_1.5, Na_V_1.8 and Na_V_1.9) are resistant to TTX and μ-conotoxins [12], [57]. Na_V_1.5 is important for heart function [58], whereas Na_V_1.8 and Na_V_1.9 are involved in the perception of pain [1]. Sequence alignment shows that all these three channels differ from Na_V_1.4 at two positions, namely, position 181 of DI and position 172 of DIV (Fig. 6). At position 181 of DI, it is a neutral asparagine in Na_V_1.4, in contrast to a positively charged lysine or arginine in TTX-resistant channels. At position 172 of DIV, it is a negatively charged glutamate in Na_V_1.4, and a neutral glutamine in TTX-resistant channels. These two residues are in close proximity to each other, as illustrated in Fig. 1B. A double mutant of Na_V_1.4 (DI-N181R, DIV-E172Q) is constructed to ascertain the role of these two residues in the selectivity of PIIIA for Na_V_1.4. We show that the mutation causes the *K*_d_ value for the binding of PIIIA to the channel to increase by two orders of magnitude, suggesting the importance of residues DI-Asn181 and DIV-Glu172 for the selectivity of PIIIA for Na_V_1.4 over TTX-resistant Na_V_ channels.

Computational mutagenesis is performed on the PIIIA-Na_V_1.4 complex in which toxin Arg14 protrudes into the filter of the channel. DI-Asn181 and DIV-Glu172 are replaced with an arginine and a glutamine, respectively, resulting in a net loss of -2 *e* in charge to the outer vestibule of the channel. Considering that PIIIA is positively charged, the mutant channel is expected to have a lower affinity for the toxin. Two Na^+^ ions are removed from the bulk to maintain charge neutrality. The complex is equilibrated for 10 ns in an unbiased MD simulation. The salt bridge Lys9-Asp161 is broken but an equivalent salt bridge, Arg20-Glu187, is formed after the equilibration. Although the same number of salt bridges is present in the structures of PIIIA in complex with the wild type and mutant Na_V_1.4, the binding affinity is found to decrease substantially after the mutation. Thus, the two residues mutated influence the binding affinity primarily via long-range electrostatic interactions with the toxin, and should have similar effects on different binding modes of the toxin and the channel. The PMF profile for the mutant channel, 12 *kT* in depth, predicts a *K*_d_ value of 20 μM (Fig. 5). The mutant Na_V_1.4 binds PIIIA about 100 fold less effectively than the wild type, suggesting that the two residues (DI-Asn181 and DIV-Glu172) are important for toxin selectivity.

## Conclusions

In this work the binding of μ-conotoxin PIIIA to a homology model of the voltage-gated sodium channel Na_V_1.4 is examined using MD simulations. Six binding modes, in which any of the six basic residues carried by PIIIA protrudes into the selectivity filter of Na_V_1.4, are considered. In all the binding modes the toxin-channel complex is stabilized by three or four salt bridges. PMF calculations predict *K*_d_ values of 300 and 150 nM for the Lys9 and Arg14 modes, respectively, in close proximity to the experimental values of 36–200 nM [12], [23], [30]. Thus, our simulations suggest that PIIIA can inhibit Na_V_1.4 with multiple alternative binding modes of similar energetics. Further studies would be required to determine whether or not the multiple-binding-mode mechanism of PIIIA can be generalized to other μ-conotoxins such as GIIIA, for which one predominant binding mode to Na_V_1.4 has been suggested [59], [60].

The multiple binding modes of PIIIA to Na_V_1.4 uncovered here is consistent with the extensive mutagenesis experiments of McArthur et al. [24]. For example, it was found that the mutation of Arg14 to an alanine or glutamate reduces the affinity of PIIIA by only 11 fold, corresponding to a change of 2.4 *kT* in the binding free energy, substantially lower than the free energy of 6–7 *kT* for a typical salt bridge [25], [26]. In addition, the alanine mutation of several other basic residues, such as Arg2, Arg12 and Lys17, causes similar reduction in the toxin's binding affinity [23], [24]. The substitution of Lys8 in GIIIA, which corresponds to Lys9 in PIIIA, to a glutamine, also causes the toxin affinity to decrease by only 7 fold [10]. The similar effect of different mutations observed experimentally support a multiple-binding-mode mechanism by PIIIA. When a basic residue of the toxin is mutated to an alanine, only one of the several binding modes is disrupted. As such each charge-changing mutation causes a similar reduction in binding affinity by the removal of a positive charge. Different isomers of PIIIA having similar affinities for Na_V_1.4 may adopt distinct binding modes to the channel [30], suggesting that a unique binding mode is inadequate to describe PIIIA binding to Na_V_ channels.

Experimental studies have found that several residues from DII and DIII of Na_V_ channels are important for the specificity of μ-conotoxins [18], [61], [62]. Experimental data have also suggested that DI-Asn181 might be involved in μ-conotoxin specificity [63]. Here it is shown that residues DI-Asn181 and DIV-Glu172 are important for the selectivity of PIIIA for Na_V_1.4 over TTX-resistant Na_V_ channels. As DI-Asn181 is replaced by a basic residue in TTX-resistant channels, one or more acidic residues may be introduced into the toxin to improve the affinity of the toxin for these channels. In fact, all known μ-conotoxins such as SmIIIA, KIIIA and BuIIIA with a *K*_d_ value for Na_V_1.5 in the micromolar range carry one or more acidic residues [4], [12].

## Supporting Information

Figure S1 **Primary structure of human Na_V_1.4.** The selectivity filter region is in purple and other regions of the pore domain included in homology modeling are highlighted in yellow. Regions of the pore domain not modeled are in grey.
(TIF)

Figure S2 **(A) Structural alignment between Na_V_Ab (red) and the four domains of our Na_V_1.4 model (blue).** (B) The position of Na_V_1.4 model (purple ribbons) relative to the lipid bilayer viewed perpendicular to the bilayer normal is shown on the left. The view along the bilayer normal from the extracellular side is shown on the right (for channel protein color scheme is as follows: green, polar; white, hydrophobic; blue, basic; red, acidic). Yellow spheres indicate lipid phosphorus atoms. Blue lines indicate the boundary of the simulation box. Water molecules and ions are not shown for clarity.
(TIF)

Figure S3 **Structures of toxin PIIIA (yellow ribbons) in complex with Na_V_1.4 (gray ribbons) in which the side chains of Arg2 (A), Arg12 (B), Lys17 (C) and Arg20 (D) protrude into the filter of the channel.** In (D), Arg2 from PIIIA is in close contact with DIII-Asp161. The complex structures are predicted from MD simulations biased with distance restraints.
(TIF)

Figure S4 **Block analysis of the PMF profiles for the Lys9 mode (A) and the Arg14 mode (B).**
(TIF)

File S1 **PDB coordinate files of Na_V_1.4 model and Na_V_1.4 in complex with PIIIA.**
(ZIP)
