# Supplementary figures and images for: Mechanism of μ-Conotoxin PIIIA Binding to the Voltage-Gated Na+ Channel NaV1.4
Source: PLoS One. 2014 Mar 27;9(3):e93267. doi: 10.1371/journal.pone.0093267 (PMC3968119; doi:10.1371/journal.pone.0093267)

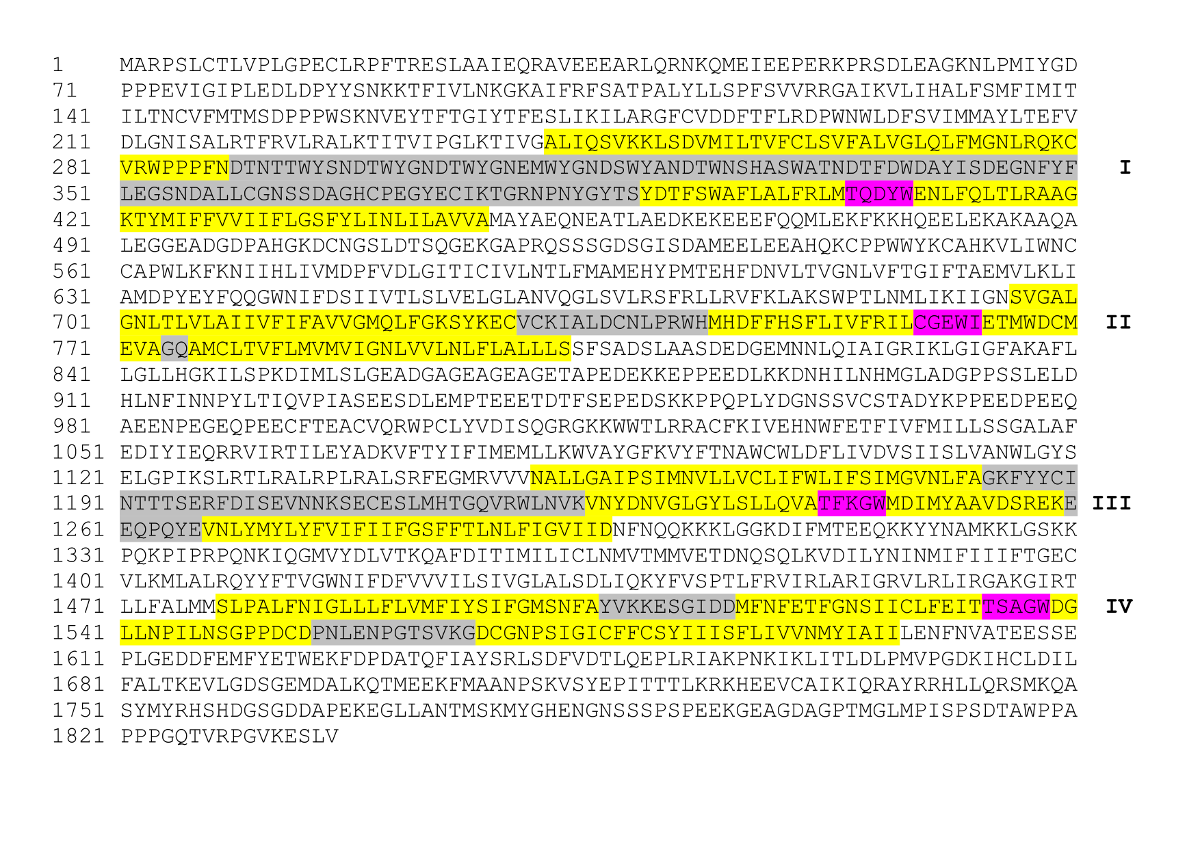

Supplement: Figure S1 — Primary structure of human NaV1.4. The selectivity filter region is in purple and other regions of the pore domain included in homology modeling are highlighted in yellow. Regions of the pore domain not modeled are in grey. (TIF) [file pone.0093267.s001.tif]

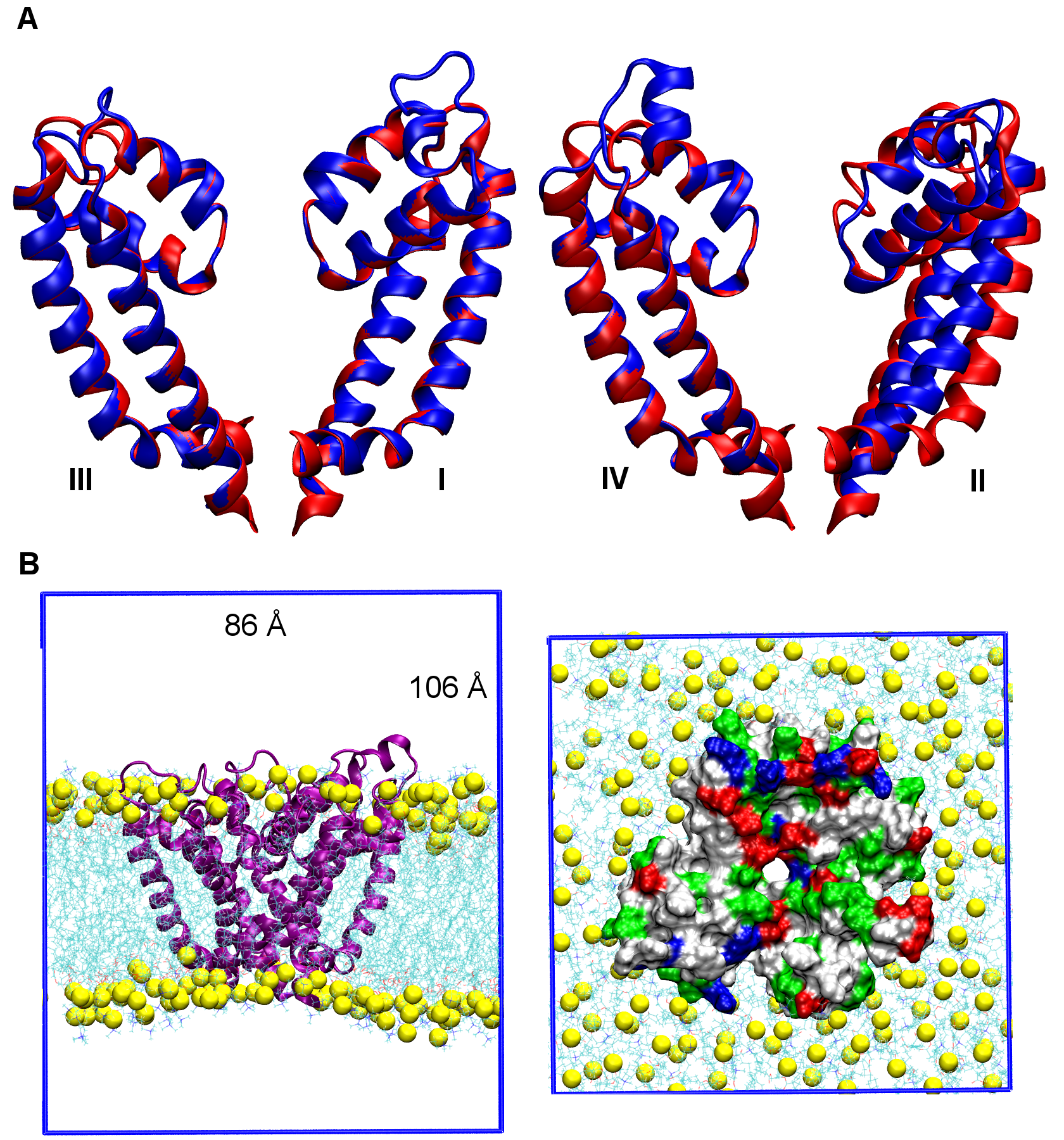

Supplement: Figure S2 — (A) Structural alignment between NaVAb (red) and the four domains of our NaV1.4 model (blue). (B) The position of NaV1.4 model (purple ribbons) relative to the lipid bilayer viewed perpendicular to the bilayer normal is shown on the left. The view along the bilayer normal from the extracellular side is shown on the right (for channel protein color scheme is as follows: green, polar; white, hydrophobic; blue, basic; red, acidic). Yellow spheres indicate lipid phosphorus atoms. Blue lines indicate the boundary of the simulation box. Water molecules and ions are not shown for clarity. (TIF) [file pone.0093267.s002.tif]

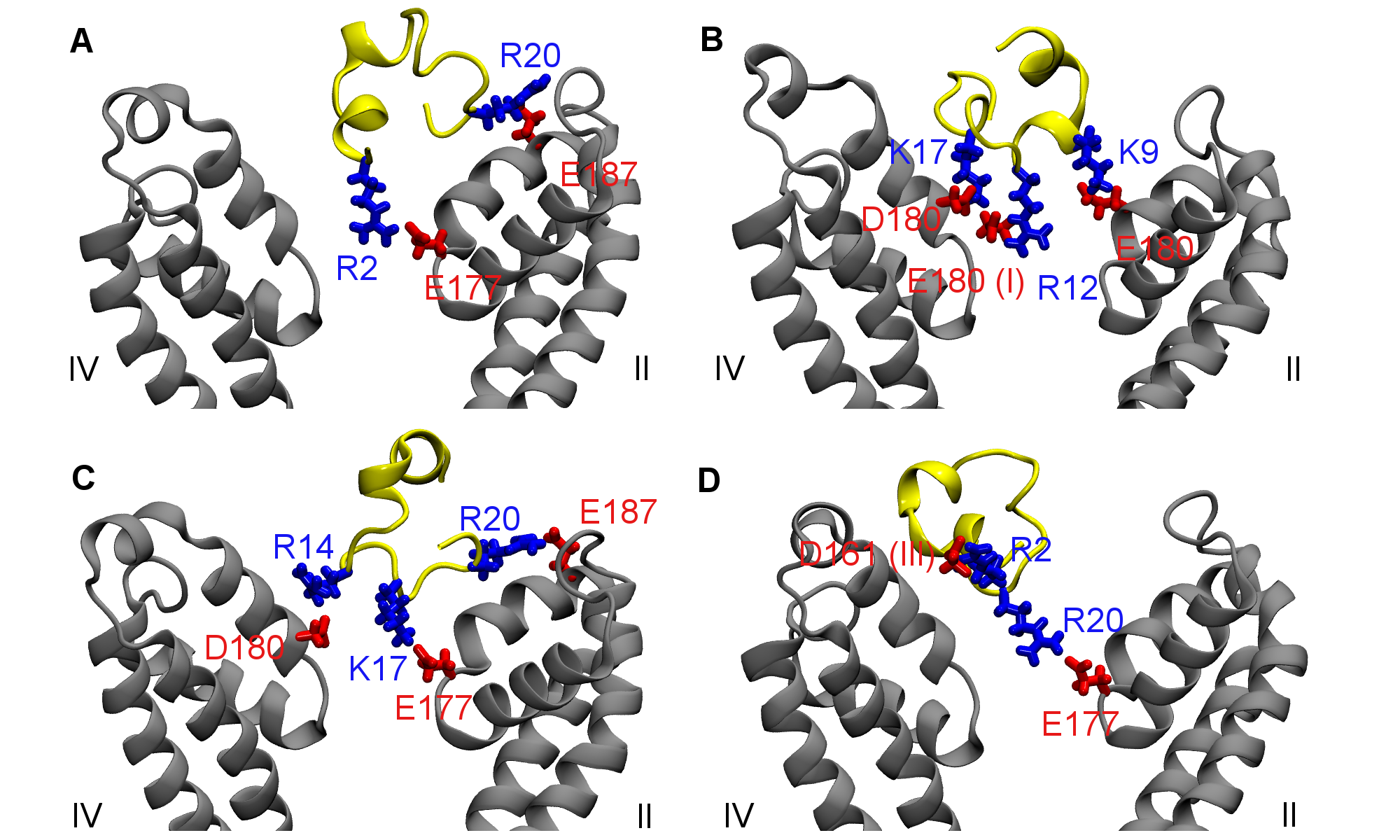

Supplement: Figure S3 — Structures of toxin PIIIA (yellow ribbons) in complex with NaV1.4 (gray ribbons) in which the side chains of Arg2 (A), Arg12 (B), Lys17 (C) and Arg20 (D) protrude into the filter of the channel. In (D), Arg2 from PIIIA is in close contact with DIII-Asp161. The complex structures are predicted from MD simulations biased with distance restraints. (TIF) [file pone.0093267.s003.tif]

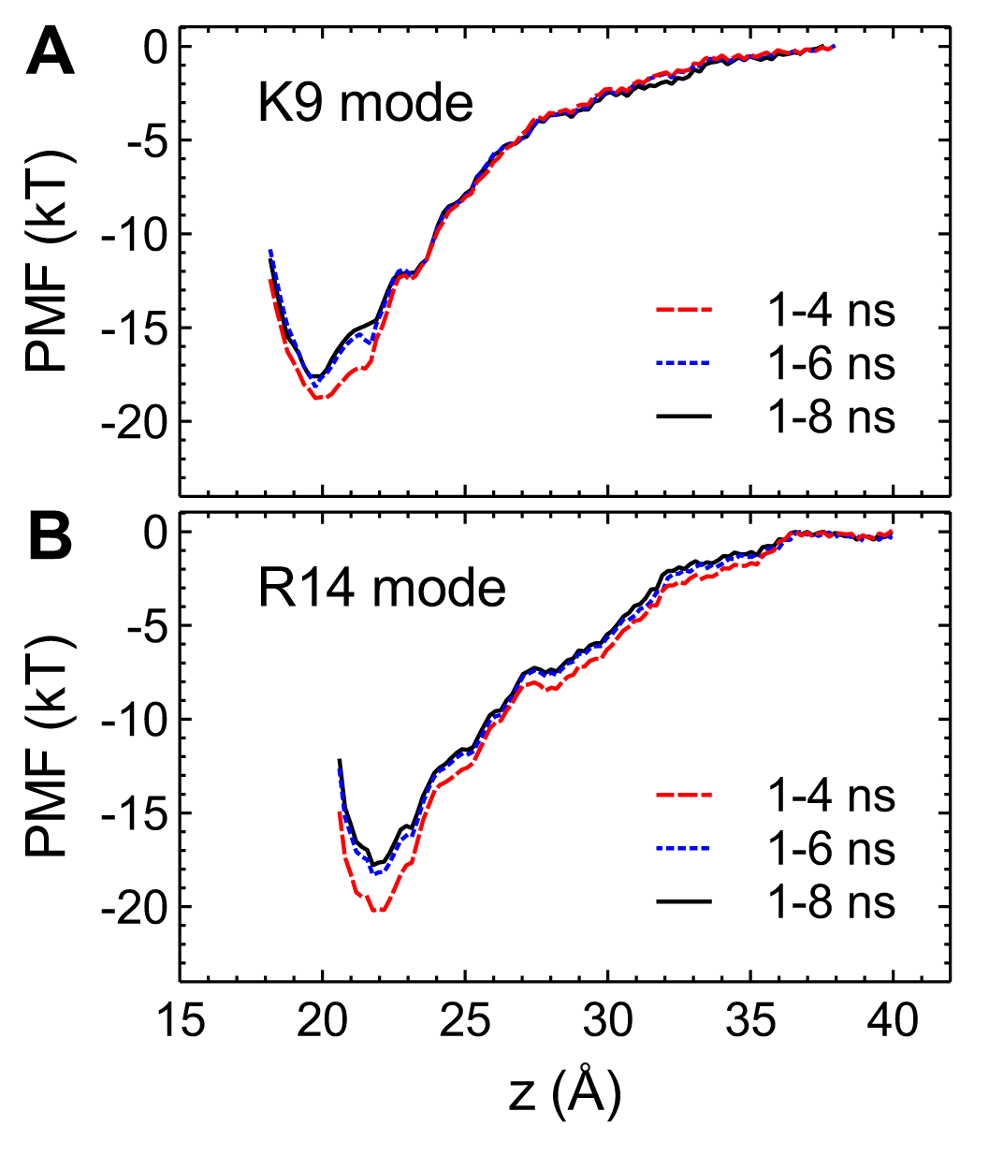

Supplement: Figure S4 — Block analysis of the PMF profiles for the Lys9 mode (A) and the Arg14 mode (B). (TIF) [file pone.0093267.s004.tif]
